# Artificial intelligence in abdominal and pelvic ultrasound imaging: current applications

**DOI:** 10.1007/s00261-024-04640-x

**Published:** 2024-11-02

**Authors:** Lie Cai, André Pfob

**Affiliations:** 1 Department of Obstetrics and Gynecology, Heidelberg University Hospital, Im Neuenheimer Feld 440, 69120 Heidelberg, Germany; 2 National Center for Tumor Diseases (NCT) and German Cancer Research Center (DKFZ), Heidelberg, Germany

**Keywords:** Ultrasound, Abdominal, Pelvic, Artificial intelligence

## Abstract

**Background:**

In recent years, the integration of artificial intelligence (AI) techniques into medical imaging has shown great potential to transform the diagnostic process. This review aims to provide a comprehensive overview of current state-of-the-art applications for AI in abdominal and pelvic ultrasound imaging.

**Methods:**

We searched the PubMed, FDA, and ClinicalTrials.gov databases for applications of AI in abdominal and pelvic ultrasound imaging.

**Results:**

A total of 128 titles were identified from the database search and were eligible for screening. After screening, 57 manuscripts were included in the final review. The main anatomical applications included multi-organ detection (n = 16, 28%), gynecology (n = 15, 26%), hepatobiliary system (n = 13, 23%), and musculoskeletal (n = 8, 14%). The main methodological applications included deep learning (n = 37, 65%), machine learning (n = 13, 23%), natural language processing (n = 5, 9%), and robots (n = 2, 4%). The majority of the studies were single-center (n = 43, 75%) and retrospective (n = 56, 98%). We identified 17 FDA approved AI ultrasound devices, with only a few being specifically used for abdominal/pelvic imaging (infertility monitoring and follicle development).

**Conclusion:**

The application of AI in abdominal/pelvic ultrasound shows promising early results for disease diagnosis, monitoring, and report refinement. However, the risk of bias remains high because very few of these applications have been prospectively validated (in multi-center studies) or have received FDA clearance.

## Introduction

In recent years, the integration of artificial intelligence (AI) techniques into medical imaging has shown promising results and may have great potential to transform the diagnostic process [1]. Among various modalities, abdominal/pelvic ultrasound imaging provides non-invasive visualization of internal organs and structures. The advancement of AI technologies has significantly enhanced the capabilities of ultrasound imaging, revolutionizing the way healthcare professionals analyze and interpret these images.

This paper aims to provide a comprehensive review of the current state-of-the-art applications of AI in abdominal and pelvic ultrasound imaging. We explored the diverse range of AI algorithms and applications tailored to address specific challenges encountered in this domain. We aimed to analyze the existing literature on AI applications in abdominal/pelvic ultrasound imaging, and to discuss the strengths, limitations, and future directions of this rapidly evolving field.

## Methods

We searched the PubMed database using the combination of the keywords:

((((abdominal[Title/Abstract] OR pelvic[Title/Abstract]).

AND (ultrasound[Title/Abstract])).

AND (machine learning[Title/Abstract] OR deep learning[Title/Abstract] OR artificial intelligence[Title/Abstract] OR intelligent[Title/Abstract] OR natural language processing[Title/Abstract]))).

NOT (Review[Publication Type]).

Each article was screened by two reviewers for inclusion or exclusion. All peer-reviewed, English-language papers from 2013 to 2024 were eligible. Editorials, letters to the editor, narrative or systematic review papers, and abstracts were excluded. Articles without available full text were excluded. As the review mainly focuses on abdominal/pelvic ultrasound, articles specific to fetal health monitoring were excluded. Reference lists of included papers were hand-searched by one reviewer and included if the inclusion criteria were met.

Besides the narrative review of published articles, we reviewed the Food and Drug Administration (FDA) approved AI devices through their official report (updated on October 2023), [2] we also reviewed ongoing relevant clinical trials from ClinicalTrials.gov database using the following combination of keywords:

Artificial intelligence OR machine learning OR deep learning.

AND abdominal OR pelvic.

AND ultrasound.

## Results

**Fig. 1:**
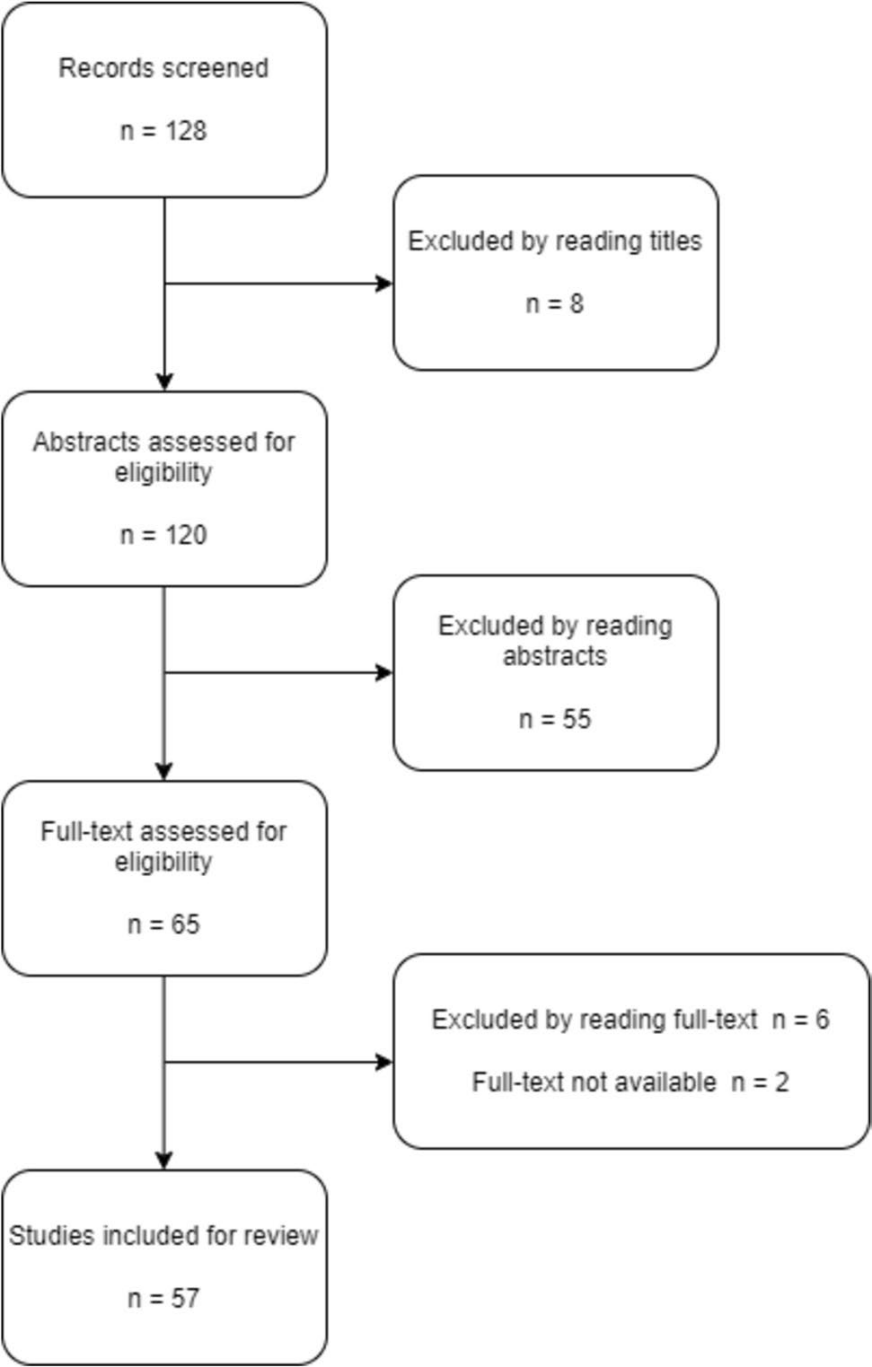
Study selection process for review

A total of 128 titles were identified from the database search and were eligible for screening. Of these, 8 studies were excluded after reading the titles, 55 studies were excluded after reading abstracts, 6 studies were excluded after reading the full-text, and 2 studies were excluded because of the unavailable full-text manuscript. A total of 57 manuscripts were included in the final review. (Fig. 1).

**Fig. 2:**
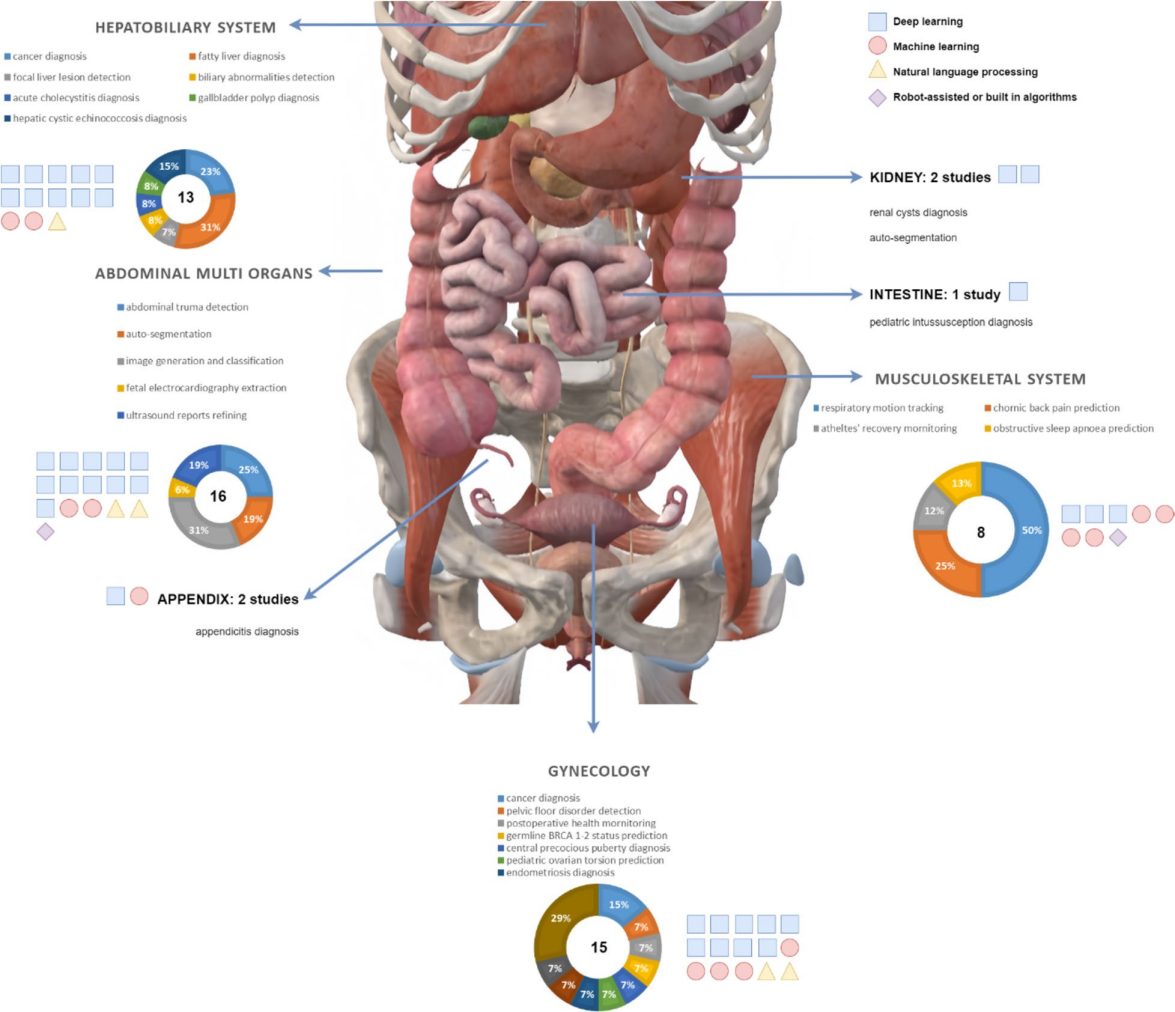
Anatomical overview of included studies according to organ systems

Based on the 57 included full-text articles and an additional search for FDA-approved devices and ongoing clinical trials, we illustrated the studies in the following categories: (Fig. 2).

(1) Hepatobiliary system;

(2) Abdominal multi-organs;

(3) Musculoskeletal system;

(4) Gynecology/pelvic;

(5) Natural language processing (NLP) for reports refining;

(6) New technologies;

(7) Image processing;

(8) FDA-approved devices;

(9) Current ongoing relevant clinical trials.

## Hepatobiliary system

The most common application of AI in ultrasound imaging of the hepatobiliary system is diagnosing liver steatosis. One study with the largest single-center dataset [3] included 21,855 abdominal ultrasound images from 2020 patients. Performance is reported with an area under the curve (AUC) of 0.996 to differentiate clinically abnormal steatosis *vs.* clinically normal. Cha DI et al. developed a convolutional neural network (CNN, a type of deep learning model, specifically designed for processing structured grid-like data) model to measure the hepatorenal index (HRI) quantitatively [4], compared to the evaluation performance with two experienced radiologists with intraclass correlation coefficients (ICCs) of 0.919, 0.916, and 0.734, respectively, without an independent validation set. Yang Y et al. found that clinical variables (age, sex, body mass index, diabetes, fibrosis-4 index, android fat ratio, and skeletal muscle via dual-energy X-ray absorptiometry) have no significant influence on neural network models [5]. They included 928 patients and developed a two-section neural network with an AUC of 0.880 for classifying non-alcoholic fatty liver severity.

Another common use case is cancer diagnosis. However, these studies’ samples are relatively small. Oezdemir I et al. developed a distance-weighted discrimination algorithm for KI-67 status prediction of hepatocellular cancer [6], an algorithm that is similar to the support vector machine (SVM, is a supervised machine learning algorithm primarily used for classification), with only 36 patients, and one of them being the validation case, it achieved an accuracy of 86% in the development set. Qian H et al. developed a machine learning model based on radiomics features acquired manually from experienced radiologists [7], for treatment response prediction of hepatocellular carcinoma transarterial chemoembolization. They included 118 patients, with intratumor and peritumor radiomics features, and the combined logistic regression (LR) model achieved the best performance with an AUC of 0.870. Karako K et al. developed a faster-region-based CNN using one patient’s intraoperative ultrasound images for liver tumor detection [8], it achieved better performance than experienced physicians with an average mean precision of 0.549 and a mean sensitivity of 0.627 in the development set.

For other common diseases in the hepatobiliary system, Yu CJ et al. developed deep-learning models for diagnosing acute cholecystitis using a large single-center dataset [9], including 10,971 ultrasound images from 7348 patients, the ResNet-50 (a deep neural network model belonging to the Residual Networks family, it has 50 layers) and MobileNet V2 (a convolutional neural network (CNN) architecture designed primarily for mobile and resource-constrained environments, it’s an improved version from MobileNet V1) achieved AUCs of 0.92 and 0.94 in the validation set, respectively. Intharah T et al. developed a CNN model for biliary abnormalities detection [10], using 6569 images from 817 patients, retrieved from the Cholangiocarcinoma Screening and Care Program (CASCAP) dataset. They used this model to assist four groups of expert-level radiologists, and this model significantly improved the performance of all radiologists, with mean accuracy from 0.50 to 0.74, and mean precision from 0.46 to 0.61. Dadoun H et al. developed a Detection Transformer (DETR) model using multi-center data for focal liver lesions detection [11], including 2551 images from 1026 patients in the development set and 155 images from 48 patients in the validation set, DETR achieved the best performance with a specificity of 90% and sensitivity of 97%, significantly better than two experts. Kim T et al. developed an ensemble model by ResNet-152, Inception v3 (a deep convolutional neural network architecture that is part of the Inception family, it has 3rd version of the inception architecture), and DenseNet-161 (a deep neural network model belonging to the Densely Connected Networks family, it has 161 layers) for diagnosing gallbladder polyps [12]. They included multi-center data of 1460 images from 501 patients and the ensemble model achieved an AUC of 0.908, accuracy 87.61%, specificity 88.35% in fivefold cross-validation.

Researchers have also put efforts into diagnosing hepatic cystic echinococcosis. Wu M et al. developed algorithms using 1,820 images from 967 patients [13]. VGG-19 (a deep convolutional neural network architecture from the Visual Geometry Group family, it has 19 layers) performed best with an accuracy of 90.6% in fivefold cross-validation. Wang Z et al. developed a normal ensemble approach (NEA) stacking model from VGG-19, ResNet-18, ViT-Base (a model from the Vision Transformer family), and ConvNeXt-T (the smallest deep convolutional neural network architecture from the ConvNeXt family, T means tiny) models [14] including 3,083 images from 972 patients. The stacking ensemble model achieved an AUC of 0.971 in the validation set, while the stacking NEA model’s AUC was 0.948.

## Abdominal multi-organs

For abdominal multi-organs, the most common application of AI in ultrasound is trauma detection. One of the largest multi-center studies aimed to acquire pediatric Focused Assessment with Sonography for Trauma (FAST) views and included 699 patients with 4925 videos and 1,062,612 images [15]. They developed a ResNet-152 model with an overall accuracy of 97.8% for videos and 93.5% for images, specifically 95.2% for abdominal upper quadrant view, 96.0% for suprapubic view. Hernandez-Torres SI et al. developed a pre-trained deep learning ShrapML model for detecting trauma in enhanced FAST views [16], and achieved over 97.0% accuracy in detecting abdominal hemorrhage, hemothorax, and negative, respectively. Leo MM et al. included 94 patients with FAST exams to build a YOLO v3 (a real-time object detection model that builds upon previous versions of You Only Look Once) model [17], achieved an AUC of 0.97 in fivefold cross-validation, but without separate validation. Sjogren AR et al. developed a radiomics machine learning study [18]. Twenty patients were included in developing a SVM model, which achieved a sensitivity of 100% and specificity of 90.0% in a tenfold cross-validation without separate validation.

Another main use case is appendicitis diagnosis. Stiel C et al. developed a random forest model using multi-center data to develop a new appendicitis AI score based on four classic appendicitis scores: Heidelberg Appendicitis Score, Alvarado Score, Pediatric Appendicitis Score, and Tzanakis Score [19]. It achieved a positive predictive value (PPV) of 95.0% and negative predictive value (NPV) of 70.0% for simple appendicitis, and PPV of 34.4%, and an NPV of 93.8% for perforated appendicitis. Marcinkevičs R et al. developed a semi-supervised extension of the multi-views concept bottleneck model (MVCBM) [20], an auto-encoder using 579 pediatric patients with 1709 images, achieved an AUC of 0.80 and area under precision-recall (AUPR) of 0.92 in the validation set.

Kanauchi Y et al. developed a YOLO v5 model using 2664 images from 1444 patients for renal cyst diagnosis [21], achieving comparable performance with two experienced radiologists with a precision of 0.85 and recall of 0.86. Li Z et al. developed a region convolutional neural network (RCNN) model for pediatric intussusception diagnosis [22]. They included 440 cases with 2325 images were included, with accuracy, recall, and specificity of 93.0%, 92.0%, and 94.1%, respectively, but 20% positive cases from the training set were shared to test set, causing data leakage. A multi-center large dataset study conducted by Zhong W et al. included two public datasets to extract fetal electrocardiography from pregnant women’s electrocardiography [23]. The fetal electrocardiogram (ECG) synthetic database (FECGSYNDB) was used for training, 2475 from PhysioNet/Computing in cardiology challenge database (PCDB) and the abdominal and direct fetal electrocardiogram database (ADFECGDB) were used for validation, they developed an auto-encoder based deep learning model, achieving a sensitivity of 96.1% and PPV of 92.3% in ADFECGDB, and a sensitivity of 92.6% and PPV of 94.6% in PCDB.

## Musculoskeletal system

Many studies have developed AI models using abdominal ultrasound images for respiratory motion tracking. Respiratory motion is produced by the coordinated movement of the chest, diaphragm, and abdominal muscles. Huang P et al. conducted a multicenter study, using 57 features acquired from 27 abdominal ultrasound sequences in a free-breathing state [24], using principal component analysis and slow feature analysis (SFA) for feature extraction, they found the most similar tracking motion by K-nearest neighbors (KNN, a simple, yet powerful, supervised machine learning algorithm used for both classification and regression tasks), achieving a mean error of 1.14 ± 1.42 mm. Mezheritsky T et al. developed an auto-encoder-based CNN model [25], that included 20 healthy volunteers for classifying mid-inhale, inhale, and mid-exhale respiratory phases. It achieved a mean error of 3.5 ± 2.4 mm. Yao C et al. developed a CNN for extracting features [26], including 58 individuals with liver respiratory motion data captured from clinical ultrasound images. It outperforms the reference model up to 76.5%. Chen A et al. used a wireless wearable sensor composed of chest and abdominal walls ultrasound to receive distance-elapsed attenuated ultrasound waves [27], including 11 cases with 1200 slices per case. They developed three random forest (RF, a supervised machine learning algorithm that is commonly used for both classification and regression tasks) algorithms, the individual RF achieved the best performance with an accuracy of 98.9%. In addition to normal respiratory motion tracking, Molnár V et al. developed an RF model for predicting obstructive sleep apnoea [28]. They included 100 patients, measuring neck, chest, and abdomen subcutaneous adipose tissues using ultrasound images, and it achieved a precision of 97.0% in the development set without further validation.

Petrovsky DV et al. documented abdominal cavity and pelvic organs ultrasound exam records from 3661 athletes for monitoring their sports recovery [29]. They developed RF and generalized linear model (GLM, a flexible extension of the linear regression model that allows for response variables to have error distributions other than the normal distribution) for classifying catabolism, anabolism, and liver metabolism, the RF achieved the best performance with an accuracy of 99.0% and a recall of 98.0%. Saleh A. et al. developed a deep learning model based on the fully convolutional networks (FCN-8, which is a type of deep learning architecture used primarily for semantic segmentation tasks, the final segmentation map is upsampled by a factor of 8) model’s backbone for measuring abdominal muscle thickness for back pain monitoring [30]. They included 400 abdominal ultrasound images and achieved a mean absolute error of 0.31 by tenfold cross-validation (Tables 1, 2, 3, 4, 5, 6).

**Table 1:** The application of AI in the ultrasound of the hepatobiliary system

Author name	Year	Single or Multicenter	Algorithm type	Specific study Aim	Outcome/endpoint
Oezdemir I [6]	2020	Single center	Machine learning	Hepatocellular carcinoma transarterial chemoembolization treatment response prediction	Accuracy: 86%; sensitivity: 89%; specificity: 82%
Yu CJ [9]	2021	Single center	Deep learning	Acute cholecystitis diagnosis	AUC: 0.940
Chou TH [3]	2021	Single center	Deep learning	Nonalcoholic fatty liver disease diagnosis	AUC: 0.996
Cha DI [4]	2021	Single center	Deep learning	Quantitative liver steatosis diagnosis	ICC: 0.919
Kim T [12]	2021	Multicenter	Deep learning	Gallbladder polyp diagnosis	AUC: 0.908, accuracy: 88%; specificity: 88%
Karako K [8]	2022	Single center	Deep learning	Intraoperative liver tumor detection	Precision: 0.549; sensitivity: 0.627
Wu M [13]	2022	Single center	Deep learning	Hepatic cystic echinococcosis diagnosis	Accuracy: 91%
Dadoun H [11]	2022	Multicenter	Deep learning	Focal liver lesions detection	Specificity: 90%, sensitivity: 97%
Qian H [7]	2023	Single center	Machine learning	Ki-67 prediction of hepatocellular cancer	AUC: 0.870
Yang Y [5]	2023	Single center	Deep learning	Nonalcoholic fatty liver disease diagnosis	AUC: 0.880
Intharah T [10]	2023	Multicenter	Deep learning	Biliary abnormalities detection	Accuracy: 0.74, precision: 0.61
Wang Z [14]	2023	Single center	Deep learning	Hepatic cystic echinococcosis diagnosis	AUC: 0.971

*AUC* area under the curve, *ICC* intraclass correlation coefficients

**Table 2:** The application of AI in the ultrasound of the abdominal multi-organs

Author name	Year	Single or multicenter	Algorithm type	Specific study Aim	Outcome/endpoint
Sjogren AR [18]	2016	Single center	Machine learning	Abdominal trauma detection	Sensitivity: 100%, specificity: 90%
Zhong W [23]	2019	Multicenter	Deep learning	Fetal electrocardiography extraction	Sensitivity: 92.60%, PPV 94.68%; F1 93.62%
Stiel C [19]	2020	Multicenter	Machine learning	Appendicitis diagnosis	For simple appendicitis (PPV 95.0%, NPV 70.0%) and very good for perforated appendicitis (PPV 34.4%, NPV 93.8%)
Kornblith AE [15]	2022	Multicenter	Deep learning	Abdominal trauma detection	Overall accuracy: 97.8% for videos, 93.4% for images
Li Z [22]	2022	Single center	Deep learning	Pediatric intussusception diagnosis	Accuracy 93.0%,; recall: 92.0%; specificity: 94.1%; F1 score: 93.2%
Kanauchi Y [21]	2023	Single center	Deep learning	Renal cysts diagnosis	Precision 85.0%, recall 86.0%
Leo MM [17]	2023	Single center	Deep learning	Abdominal trauma detection	AUC: 0.97; accuracy: 95.0%; sensitivity: 95.0%; specificity:94.0%
Hernandez-Torres SI [16]	2023	Single center	Deep learning	Abdominal trauma detection	Accuracy: 97.0%
Marcinkevičs R [20]	2024	Single center	Deep learning	Appendicitis diagnosis	AUC: 0.80

*AUC* the area under the curve, *PPV* positive-predictive values, *NPV* negative-predictive values

**Table 3:** The application of AI in the ultrasound of the musculoskeletal system

Author name	Year	Single or Multicenter	Algorithm type	Specific study Aim	Outcome/endpoint
Huang P [24]	2019	Multicenter	Machine learning	Respiratory motion tracking for abdominal radiation therapy	Mean error: 1.14 ± 1.42 mm
Chen A [27]	2020	Single center	Machine learning	Respiratory motion tracking	Accuracy: 98.9%
Saleh A [30]	2021	Single center	Deep learning	Measuring abdominal muscle thickness for back pain monitoring	Mean absolute error: 0.313
Petrovsky DV [29]	2022	Single center	Machine learning	Athletes recovery monitoring	Accuracy: 99.0%; recall: 98.0%
Molnár V [28]	2022	Single center	Machine learning	Obstructive sleep apnea prediction	Precision: 97.0%
Mezheritsky T [25]	2022	Single center	Deep learning	Respiratory motion tracking	Mean error: 3.5 ± 2.4 mm
Yao C [26]	2022	Single center	Deep learning	Respiratory motion tracking	Mean absolute error, compared to reference models

**Table 4:** The application of AI in the ultrasound of the gynecology

Author name	Year	Single or multicenter	Algorithm type	Specific study Aim	Outcome/endpoint
Otjen JP [36]	2020	Single center	Machine learning	Pediatric ovarian torsion prediction	AUC: 0.96; sensitivity: 95.0%; specificity: 92.0%
Nero C [33]	2020	Single center	Machine learning	Germline BRCA 1–2 status prediction	Specificity: 87.0%; NPV: 73.0%; accuracy: 72.0%
Maicas G [37]	2021	Single center	Deep learning	Pouch of Douglas (POD) for endometriosis diagnosis	AUC: 0.97; accuracy: 88.8%; sensitivity: 88.6%; specificity: 90.0%; PPV: 98.7%; NPV: 47.7%
Gao Y [31]	2022	Multicenter	Deep learning	Ovarian cancer diagnosis	AUC: 0.870 AI alone 0.876 average AI assisted 6 radiologists
Zhu Y [34]	2022	Single center	Deep learning	Pelvic rehabilitation function assessment after laparoscopic hysterectomy	Accuracy: 97.34%
Abdel Hady DA [35]	2023	Single center	Machine learning	Pelvic tilt and lumbar angle prediction	R^2^: 0.976
Chen T [38]	2023	Single center	Machine learning	Central precocious puberty detection	Sensitivity: 81.0%; specificity: 72.0%; F1 score: 0.80
Deeparani M [32]	2023	Multicenter	Deep learning	Pelvic mass detection	Accuracy: 99.8%

*AUC* area under the curve, *BRCA* breast cancer gene, *NPV* negative predictive value, *PPV* positive predictive value, *AI* artificial intelligence, *R*^2^ coefficient of determination

**Table 5:** The application of NLP algorithms in abdominal and pelvic ultrasound

Author name	Year	Single or multicenter	Specific study Aim	Outcome/endpoint
Garla V [39]	2013	Single center	Abdominal ultrasound, CT, and MRI report cancer alert detection	F1: 0.77; Sensitivity: 94.3%; PPV: 87.7%
Morioka C [40]	2016	Single center	Abdominal aortic aneurysms (AAA) report classification	Precision: 99.0%; recall: 99.0%
Cheng JJ [41]	2019	Single center	Polycystic ovary morphology classification	Accuracy: 97.6%
Yazdani A [42]	2020	Single center	Misspelling detection and correction for the Persian language	Detection up to 90.3%; accuracy for correction: 88.6%
Xie F [43]	2022	Multicenter	Preterm birth report detection	PPV: 97.0%;

*PPV* positive predictive value

**Table 6:** The application of AI for image processing in abdominal and pelvic ultrasound

Author name	Year	Single or multicenter	Prospective or Retrospective	Algorithm type	Specific study Aim	Outcome/endpoint
Kim KB [46]	2015	Multicenter	Retrospective	Deep learning	Auto-segmentation	Accuracy: 95.0%
Cheng PM [52]	2017	Multicenter	Retrospective	Deep learning	Image classification	Accuracy: 90.4%%
Gibson E [56]	2018	Multicenter	Retrospective	Deep learning	Image generation	Exemplary images
Chen L [53]	2019	Multicenter	Retrospective	Deep learning	Image classification	Precision: 89.4%; recall: 89.7%; F1: 89.4%
Camps SM [55]	2020	Multicenter	Retrospective	Deep learning	Image classification	Accuracy: 94.0%; specificity: 95.0%; sensitivity: 92.0%
Vitale S [57]	2020	Single center	Retrospective	Deep learning	Image generation	Exemplary images
Lei Y [47]	2021	Single center	Retrospective	Deep learning	Auto-segmentation	DSC: 94.0%; HD_95_: 1.65 mm
Housden J [44]	2021	Single center	Prospective	Robot-assisted	Image generation	Image quality assessment: 95%;
Ashkani Chenarlogh V [48]	2022	Multicenter	Retrospective	Deep learning	Auto-segmentation	DSC: 97.6%
Singh VK [49]	2022	Single center	Retrospective	Deep learning	Auto-segmentation	DSC: 90.0%
Mezheritsky T [25]	2022	Single center	Retrospective	Deep learning	Respiratory motion track; real-time target tracking	Mean tracking error: 3.5 mm
Cammarasana S [58]	2023	Single center	Retrospective	Deep learning	Image generation	PSNR: 4.4%
Szentimrey Z [50]	2023	Single center	Retrospective	Deep learning	Auto-segmentation	DSC: 87.4%
Peng T [51]	2023	Single center	Retrospective	Deep learning	Auto-segmentation	DSC: 94.6%
Lawley A [54]	2024	Single center	Retrospective	Deep learning	Image classification	Accuracy: 95.1%

*DSC* mean dice score, *HD*_95_, Hausdorff distance, *PSNR* peak signal-to-noise ratio

## Gynecology

The main application of AI in pelvic/transvaginal ultrasound images is cancer diagnosis. Gao Y et al. conducted a large multicenter dataset study for ovarian cancer diagnosis [31], including 34,488 ultrasound images of 3755 patients with ovarian cancer and 541,442 images of 101,777 controls from 10 hospitals. They developed a CNN model and achieved the best AUC of 0.88 with CNN assisted evaluation by experienced radiologists. Deeparani M et al. conducted a large multicenter dataset study for pelvic mass detection [32], including 23,965 ultrasound pelvic mass images for training, and 15,977 ultrasound pelvic mass images for validation. They developed various deep learning models, the CNN model achieved the best performance with an accuracy of 99.8%. Nero C et al. developed various machine learning models based on ultrasound radiomics features for germline (Breast cancer gene) BRCA 1–2 status prediction [33], they included 255 patients, and the auto-machine-learning achieved the best performance with a specificity of 87.0%, a NPV of 73.0%, and accuracy of 72.0%.

Another main application is the pelvic rehabilitation function assessment. Zhu Y et al. developed a bilinear CNN model for classifying pain degrees after laparoscopic hysterectomy [34], achieved the highest accuracy of 97.3% with 80 patients in the development set. Abdel Hady DA et al. developed various machine learning models for pelvic tilt and lumbar angle prediction [35], which is a key factor in assessing pelvic function. They included 92 patients, and the AdaBoost model achieved the best performance with an R^2^ (coefficient of determination) of 0.98 by cross-validation.

Otjen JP et al. developed a decision tree model for pediatric ovarian torsion prediction [36]. They included 450 patients, and the model achieved an AUC of 0.96 in the development set without separate validation. Maicas G et al. developed a ResNet-based model for pouch of Douglas (POD) detection, which is a key sign for endometriosis diagnosis [37]. They included 749 videos and achieved an AUC of 0.97 in the validation set. Chen T et al. developed various machine learning models for central precocious puberty detection [38]. They included 455 patients, and the eXtreme Gradient Boosting (XGBoost, a powerful, scalable, and efficient machine learning algorithm that is widely used for supervised learning tasks) model performed the best with a sensitivity of 81.0%, a specificity of 72.0%, and a F1 score of 0.80.

In addition to AI application for disease diagnosis and health monitoring, AI also greatly enhanced the application of abdominal ultrasound techniques, including natural language processing (NLP, a field of artificial intelligence that focuses on the interaction between computers and humans through natural language. It enables machines to understand, interpret, and generate human language in a meaningful and useful way) for ultrasound reports refining, and new technologies in image processing.

### NLP for reports refining

Garla V et al. developed a Laplacian text-based SVM model for abdominal ultrasound, CT, and MRI reports for cancer alert detection [39]. They used 19,485 randomly sampled unlabeled notes in addition to the training reference standards, evaluated on 520 labeled reports. The algorithms achieved a PPV of 87.7%, sensitivity of 94.3%, and a F1 score of 0.77. Morioka C et al. developed a text-based feature vector with a decision table classifier algorithm with General Architecture for Text Engineering (GATE) development system for abdominal aortic aneurysms (AAA) reports classification [40]. They included 1,402 ultrasound reports and achieved a precision of 99.0% and a recall of 99.0% without separate validation set. Cheng JJ et al. developed a rule-based classifier for polycystic ovary morphology classification [41]. They included 39,093 reports from 25,535 women and achieved an accuracy of 97.6% in the validation set. Yazdani A et al. developed a N-gram model for misspelling detection and correction for the Persian language in abdominal and pelvic ultrasound reports [42]. They included 3531 reports containing 106,084 words, and 1,509 misspellings for training, and 428 reports containing 19,264 words and 187 misspelling for validation, the N-gram algorithm detected 90.3% misspellings and achieved accuracy for correction of 88.6%. Xie F et al. conducted a multicenter study using the NLP algorithm for preterm birth reports detection [43]. They included 441,673 patients, 103,139 patients with preterm births, and the algorithm achieved a PPV of 97.0% in the separate validation set.

### New technologies

Ultrasound examination is vulnerable to the operator's performance using the transducer, and the reproducibility of the obtained image can be an issue. Housden J et al. conducted a prospective study with a dual probe robot to acquire standardized ultrasound images [44]. The robot has 17 degrees of freedom with two arms to hold and control two probes, and it achieved 95% of good or acceptable ultrasound image quality compared to human experts (100%).

Chronic back pain has a high prevalence and seriously affects the patient’s quality of life. Perotti L et al. used a wearable intelligent ultrasound device to monitor abdominal muscle activity in real-time [45], providing visual biofeedback to patients and physicians. They included 15 chronic back pain patients who wore this device for two hours and then finished questionnaires and scales to evaluate their experience and acceptance of it. They reported a high willingness to use the system as a feedback tool both in physiotherapeutic practices and at home. The automated detection and evaluation of muscle contraction states were highlighted as a major benefit of the system compared to the more subjective feedback provided by traditional methods such as palpation.

### Image processing

The main application of AI in ultrasound image processing is auto-segmentation. Auto-segmentation means to delineate the area of interest (ROI) from images automatically for further research. Kim KB et al. conducted a step-by-step pipeline to extract the outline of the appendix for quantitative measurement [46], including binarization, interpolation, removal of noise, and a fuzzy Adaptive Resonance Theory (ART, a type of neural network model used for unsupervised learning and clustering.) algorithm. Their method achieved an accuracy of 95.0% (38 of 40) without separate validation. Lei Y et al. developed a CNN based on the backbone of a fully convolutional one-state object detector (FCOS) [47], to auto-segment pelvic organs. They included 83 cases, and among multiple pelvic organs, the model achieved a best Dice similarity coefficient (DSC) of 0.94 in segmenting prostate, and a best 95% Hausdorff distance (HD_95_) of 1.65 mm in segmenting rectum in fivefold cross-validation. Ashkani Chenarlogh V et al. developed a U-Net-based algorithm to auto-segment fetal head and abdominal circumferences from pregnant women’s abdominal ultrasound images [48]. They included 694 patients in total and obtained DSC and Jaccard coefficients of 97.6% and 95.4% for fetal head segmentation; 95.07%, and 91.99% for fetal abdominal segmentation; and 97.5%, and 95.0% on the public HC18-Grand challenge dataset. Singh VK et al. developed a U-Net-based model for segmenting ovaries and antral follicles [49]. They included 197 patients, and the model achieved a DSC of 90.0% for ovaries and 81.0% for antral follicles. Szentimrey Z et al. developed a U-Net-based model for segmenting pelvic multi-organs [50], including 248 volumes from 135 patients, and the model achieved a DSC of 87.4% in a separate validation set. Peng T et al. developed a CNN model for segmenting kidneys [51]. They included 1380 images from 115 patients, and the model achieved a DSC of 94.6%.

Another main application is image classification. Cheng PM et al. developed a CaffeNet-based model to classify abdominal ultrasound images into 11 categories [52]. They included 4094 images from 136 studies as the training set, and 1423 images from 49 studies as the validation set. The model achieved an accuracy of 90.4% in the validation set. Chen L et al. developed a CNN model for image classification for various tasks [53], and tested its performance by classifying 13 categories of fetal scan planes in 2694 pregnant abdominal ultrasound images. The model achieved a precision of 89.4%, a recall of 89.7%, and a F1 score of 89.4%. Lawley A et al. developed various deep learning models to classify routine upper abdominal ultrasound scans into 16 categories [54], and validated models’ performance on 26,294 images. The InceptionV3 model performed the best with an accuracy of 95.1%. Camps SM et al. developed a DenseNet-based model [55], they included three different datasets with 11,148 images from 36 patients. The model achieved an accuracy of 94.0%, a specificity of 95.0%, and a sensitivity of 92.0%.

Learning the characteristics of ultrasound images and then generating specified views of ultrasound images is also a promising application. Gibson E et al. used a built-in application in Tensorflow to develop an intelligent pipeline [56], including segmentation, classification, detection, registration, reconstruction, enhancement, model representation, and generation. The model demonstrated a smooth variation between different amounts of ultrasound shadow artifacts. Vitale S et al. developed a CycleGANs model with two generators [57], a U-Net (a type of convolutional neural network designed for image segmentation, especially in biomedical applications.) and a ResNet, to improve realism in abdominal ultrasound simulation from CT scans. They obtained promising realism scores evaluated by 21 technicians or physicians. Cammarasana S et al. developed a state-of-the-art DNN model for improving the quality of ultrasound images [58]. The model improved the PSNR value of 4.4% in abdominal ultrasound raw images. Mezheritsky T et al. developed an auto-encoder model to generate 3D ultrasound images through 2D abdominal images for tracking respiratory behaviors [25]. They included 20 healthy volunteers and the model achieved a mean tracking error of 3.5 mm.

## FDA approved devices

The U.S. Food and Drug Administration (FDA) has approved 692 artificial intelligence (AI) and machine learning (ML) algorithms as of October 2023 [2]. 17 of 692 were ultrasound AI devices, 16 of 17 were supported by GE Medical Systems Ultrasound and Primary Care Diagnostics company, and the other one was supported by Philips Ultrasound. All 17 devices can be implemented in abdominal ultrasound images, and their main use include measurement and analysis of the human body and fluid; specialized for cardiac imaging; diagnostic review and analysis of ultrasound images, patient record management and reporting; abdominal imaging (GYN, pelvic and infertility monitoring/follicle development); and multi-mode diagnostic imaging. Table 7 summarizes the FDA-approved AI ultrasound devices.

**Table 7:** The FDA-approved AI ultrasound devices

Date of final decision	Submission number	Device	Company	Main intend for use
05/02/2018	K180599	Venue	GE Medical Systems Ultrasound and Primary Care Diagnostics, LLC	Measurement and analysis of the human body and fluid
10/25/2018	K181685	Vivid E80, Vivid E90, Vivid E95	GE Medical Systems Ultrasound and Primary Care Diagnostics, LLC	Specialized for use in cardiac imaging
07/16/2020	K200497	Vivid S60N, Vivid S70N	GE Medical Systems Ultrasound and Primary Care Diagnostics, LLC	Cardiac imaging
09/09/2020	K200708	Vivid iq	GE Medical Systems Ultrasound and Primary Care Diagnostics, LLC	Cardiovascular and shared services
07/23/2020	K200743	Vivid E80/ Vivid E90/ Vivid E95	GE Medical Systems Ultrasound and Primary Care Diagnostics, LLC	Cardiac imaging
09/09/2020	K200851	Vivid T8, Vivid T9	GE Medical Systems Ultrasound and Primary Care Diagnostics, LLC	Diagnostic review and analysis of ultrasound images, patient record management and reporting
09/18/2020	K200852	EchoPAC Software Only, EchoPAC Plug-In	GE Medical Systems Ultrasound and Primary Care Diagnostics, LLC	Diagnostic review and analysis of ultrasound images, patient record management, and reporting
09/10/2021	K210438	Versana Premier	GE Medical Systems Ultrasound And Primary Care Diagnostics	Measurement and analysis of the human body and fluid
06/06/2022	K220358	Voluson Expert 22, Voluson Expert 20, Voluson Expert 18	GE Medical Systems Ultrasound And Primary Care Diagnostics	Abdominal (GYN, pelvic, and infertility monitoring/follicle development)
05/11/2022	K220446	Versana Balance	GE Medical Systems Ultrasound And Primary Care Diagnostics	Measurement and analysis of the human body and fluid
07/15/2022	K220619	Vivid S60N, Vivid S70N	GE Medical Systems Ultrasound And Primary Care Diagnostics	Cardiac imaging
06/21/2022	K220800	Venue Go	GE Medical Systems Ultrasound And Primary Care Diagnostics	Measurement and analysis of the human body and fluid
07/22/2022	K220882	Vivid E80, Vivid E90, Vivid E95	GE Medical Systems Ultrasound And Primary Care Diagnostics	Measurement and analysis of the human body and fluid
07/22/2022	K220940	EchoPAC Software Only, EchoPAC Plug-in	GE Medical Systems Ultrasound And Primary Care Diagnostics	Diagnostic review and analysis of ultrasound images, patient record management, and reporting
07/18/2022	K221147	Vivid T8, Vivid T9	GE Medical Systems Ultrasound And Primary Care Diagnostics	Cardiac imaging
07/18/2022	K221148	Vivid iq	GE Medical Systems Ultrasound and Primary Care Diagnostics, LLC	Cardiovascular and shared services
05/04/2023	K223771	Lumify Diagnostic Ultrasound System	Philips Ultrasound	Diagnostic imaging in B(2D), Color Doppler, Combined(B + color), Pulsed Wave Doppler(PWD), and M-modes

## Ongoing clinical trials

There are a few ongoing clinical trials on the application of AI in abdominal/pelvic ultrasound imaging. Ambroise Grandjean G et al. are conducting a prospective trial using a deep learning algorithm for extracting fetal imaging and biometry measurements from 3D ultrasound volumes automatically from abdominal ultrasound imaging (NCT03812471). Carlos Robles-M et al. intend to build a CNN algorithm that recognizes the abdominal anatomical structures during linear and radial endoscopic ultrasound evaluations (NCT05151939). Jan D et al. are conducting a single-center study using an intelligent algorithm to assess and predict pelvic floor recovery after one year of childbirth based on trans-perineal ultrasound images and patient self-reported symptoms (NCT05530681). Theo R et al. developed an algorithm that can automatically segment the pelvic vessels from ultrasound images and will validate it prospectively (NCT05637346).

## Discussion

In this review, we provide an overview of current AI applications in abdominal/pelvic ultrasound imaging. We searched and categorized studies based on anatomical locations (the hepatobiliary system, abdominal multi-organs, musculoskeletal system, and gynecology/pelvic). The current main applications of AI in abdominal ultrasound imaging include disease diagnosis (42.1%, 24 of 57), cancer diagnosis (7.0%, 4 of 57), health monitoring (15.8%, 9 of 57), fetal health monitoring (1.8% 1 of 57), NLP for ultrasound report refining (5.3%, 3 of 57), and image processing (28.1%, 16 of 57). We also demonstrated the FDA-approved AI devices in abdominal ultrasound imaging and relevant ongoing clinical trials.

AI has been widely used in abdominal/pelvic ultrasound and has shown promising results. Although relevant studies are emerging, there is still much room that needs to be improved, in methodology, prospective validation, digital infrastructure, and ethnic diversity.

First, an external or independent validation set is necessary to evaluate the models’ performance. In our review, we saw 2 studies in the hepatobiliary system [12, 13], 1 study in the musculoskeletal system [30], 1 study in gynecology [35] that didn’t use an independent validation set, they directly used cross-validation to evaluate models’ performance. This can lead to positive bias because, for k-fold cross-validation, we split the data into k parts (k-fold cross-validation), using one of the k parts for validation, and the rest of k-1 parts for training the model, the process will repeat k times. By repeating the process of cross-validation, the model is learning the inter-relationships and representative features from data and adjusting its parameters, once the cross-validation is finished, the model has already learned the features and outcomes of the data (so-called data-leakage) [59], an external validation set is required to assess the fine-tuned model. It’s not that the more complex the model, the better the performance, simpler models are easier to explain, save operating time, and have a lower risk of over-fitting (models performed well on training and internal validation but performed worse on external validation) [60]. Wang et.al [14] build VGG19, ResNet18, ViT-Base models, and a stacking model to ensemble all models above for diagnosing hepatic cystic echinococcosis, while the ensemble model showed comparable or superior performance without statistical significance.

Second, prospective validation is essential for trustworthy and reliable AI development. The multi-center setting is to increase the model’s generalizability by learning different patterns of data as much as possible. However, with the large number of studies we included, only one image processing study was prospectively validated [44], none of the disease diagnosing or health monitoring studies were prospectively validated. Only 25% (14 of 57) studies were multi-center studies. For some models developed by large single-center datasets [3, 9], prospective validation would be highly desired. The number of registered relevant clinical trials is also limited. Gulshan et al. developed a deep learning model for the detection of diabetic retinopathy, [61] and achieved AUC values of 0.99 in two separate American validation sets in retrospective settings, while the performance decreased when prospectively validated in 11 rural clinics in Thailand, [62] because the lack of dedicated screening rooms that can take high-quality images, and inconsistent broadband connectivity, and patients’ concerns about having to follow up at a hospital. During development, AI models are trained and validated on historical and existing data, like the other retrospective clinical studies, the data itself may be produced within certain biases or patterns. Prospective validation is to ensure models generalize well on unseen, real-world data, which may have new patterns, variations, and shifts that were not captured in the training data [63].

Third, in this review, none of the included studies reported the composition of ethnic groups. [62]Different ethnic groups may contain unseen trends or patterns that can be captured by AI models. [64] For example, Obermeyer et al. [65] found evidence that Black patients who were assigned the same level of risk by an algorithm were sicker compared to White patients, which reduced the number of Black patients identified for extra care by more than half. Hsu et al. developed an ensemble deep learning model for breast mammography screening [66], and validated its performance in the UCLA cohort, the Kaiser Permanente Washington cohort, and the Karolinska Institute cohort separately, found the high performance of an ensemble deep-learning model for automated screening mammography interpretation did not generalize to a more diverse screening cohort. A recent radiomics study developed an algorithm using pretreatment MRI radiomics features to predict response to neoadjuvant chemotherapy for breast cancer patients [67], the algorithm was developed in a German cohort and separately validated in the American cohort and the Chinese cohort, showing a non-inferior performance in the American cohort (AUC: 0.75 vs. 0.81, p = 0.543) but a lower performance in the Chinese cohort (AUC: 0.61 vs. 0.81, p = 0.004). A single ethical composition might lead to a positive bias in the model’s performance.

Fourth, only 5.3% (3 of 57) studies reported the computing time of their algorithms [9, 17, 21], which can be a crucial factor in some emergency cases, reporting the cost of these algorithms is also important. Digital infrastructure would be a hindrance to the application in rural areas [68]. Big data machine learning studies require a high memory of Central Processing Unit (CPU); deep learning studies, when doing image processing and training, especially data augmentation (a technique to increase the data size by flipping, rotating, and cropping images) produce images to grow exponentially, require a high memory of Graphics Processing Unit (GPU), which are expensive nowadays. When putting AI algorithms as medical devices into clinical practice, both the European Medicines Agency (EMA) and the FDA require that AI devices meet certain performance and safety standards, which could include timely delivery of results if relevant to the clinical application [69, 70]. We suggest reporting the type of CPU and GPU, the size of the memory of the equipment for developing AI algorithms, and the computing time for calculating the AUC value and making individual predictions in future studies.

Fifth, as classical guidelines for developing intelligent algorithms have been released [71], which provided transparent and rigorous suggestions to design AI studies in terms of enrollment, allocation, follow-up, and analysis. We appeal to researchers to strictly follow them to mitigate bias as much as possible.

## Conclusion

In summary, the application of AI in abdominal/pelvic ultrasound showed promising results and has great potential in updating clinical diagnosis and management. However, the risk of bias remains high because very few of these applications have been prospectively validated (in multi-center studies) so far or received FDA clearance.

## Data Availability

No datasets were generated or analysed during the current study.

## Abbreviations

**AI**: Artificial intelligence
**FDA**: Food and drug administration
**AUC**: Area under the curve
**CNN**: Convolutional neural networks
**HRI**: Hepatorenal index
**ICC**: Intraclass correlation coefficients
**SVM**: Support vector machine
**CASCP**: Cholangiocarcinoma screening and care program
**DETR**: Detection transformer
**ResNet**: Residual networks
**DenseNet**: Dense connected networks
**VGG**: Visual geometry group
**ViT Base**: Vision transformer base
**FAST**: Focused assessment with sonography for trauma
**YOLO**: You only look once
**MVCBM**: Multi-views concept bottleneck model
**AUPR**: Area under precision-recall
**RCNN**: Region convolutional neural network
**ECG**: Electro cardio gram
**FECGSYNDB**: Fetal electro cardio gram synthetic database
**PCDB**: Physionet/computing in cardiology challenge database
**ADFECGDB**: Abdominal and direct fetal electro cardio gram database
**SFA**: Slow feature analysis
**KNN**: K-nearest neighbors
**RF**: Random forest
**GLM**: Generalized linear model
**FCN**: Fully convolutional networks
**PPV**: Positive predictive value
**NPV**: Negative predictive value
**BRCA**: Breast cancer gene
**POD**: Pouch of douglas
**XGBoost**: Extreme gradient boosting
**NLP**: Natural language processing
**GATE**: General architecture for text engineering
**AAA**: Abdominal aortic aneurysms
**ROI**: Area of interest
**FCOS**: Fully convolutional one-state object detector
**DSC**: Dice similarity coefficient
**EMA**: European medicines agency
